# Supplementary material for: Mice Lacking PLAP-1/Asporin Show Alteration of Periodontal Ligament Structures and Acceleration of Bone Loss in Periodontitis
Source: Int J Mol Sci. 2023 Nov 5;24(21):15989. doi: 10.3390/ijms242115989 (PMC10649079; doi:10.3390/ijms242115989)
Supplement: Supplementary file 1 [file ijms-24-15989-s001.zip › ijms-2644249-supplementary.pdf]

Supplemental Table S1. The number of animals used in each experiments.

Figure number	Number of wild type mice	Number of *PLAP-1* KO
Figure 1B	4	4
Figure 1D	3	3
Figure 1F	4	4
Figure 1H	3	3
Figure 2A	4	4
Figure 2B	4	4
Figure 2C	3	3
Figure 3B, 3C	4	4
Figure 4B	4	4
Figure 5B	11	12
Figure 5E	6	6
